# Comparison of Culture and PCR Methods for Diagnosis of *Mycobacterium tuberculosis* in Different Clinical Specimens

**DOI:** 10.5812/jjm.8939

**Published:** 2014-02-01

**Authors:** Aida Gholoobi, Ali Masoudi-Kazemabad, Mojtaba Meshkat, Zahra Meshkat

**Affiliations:** 1 Department of Modern Sciences and Technologies, School of Medicine, Mashhad University of Medical Sciences, Mashhad, IR Iran; 2 Department of Biology, Science and Research Branch, Islamic Azad University, Tehran, IR Iran; 3 Department of Biostatistics, Mashhad Branch, Islamic Azad University, Mashhad, IR Iran; 4 Antimicrobial Resistance Research Center, Faculty of Medicine, Mashhad University of Medical Sciences, Mashhad, IR Iran

**Keywords:** *Mycobacterium tuberculosis*, Polymerase Chain Reaction (PCR), Culture, Diagnosis

## Abstract

**Background:**

Tuberculosis remains a global epidemic, especially in developing countries, including Iran. Rapid diagnosis of active *Mycobacterium tuberculosis* infection plays a critical role in controlling the spread of tuberculosis. Conventional methods may take up to several weeks or longer to produce results. In addition to multiplicity of steps involved in conventional detection, including isolation, identification and drug susceptibility testing, the slow growth rate of *M. tuberculosis* is also responsible for this lengthy time.

**Objectives:**

The aim of this study was to compare the polymerase chain reaction (PCR) and culture methods for the detection of *M. tuberculosis* in different clinical specimens.

**Materials and Methods:**

This study was performed on different samples (urine, gastric aspirate, bronchoalveolar lavage, pleural fluid, cerebrospinal fluid, ascetic fluid and joint fluid specimens) of tuberculosis suspected patients. *M. tuberculosis* DNA was extracted directly from different samples using two different protocols. Next, PCR was performed using three sets of specific primers to detect members of *Mycobacterium* genus, *M. tuberculosis* complex and non-tuberculosis Mycobacteria. The results were then compared with that of the culture method, which is considered as the gold standard method.

**Results:**

The concordance rate between the three sets of primers was calculated and IS6110/buffer PCR method showed good agreement with the LJ culture method (κ = 0.627, P < 0.0001). The sensitivity of IS6110/buffer PCR was 58.33%, with specificity of 77.78%; the positive and negative predictive values were 100% and 78.26%, respectively. Buffer method for DNA extraction was proved to give a higher accuracy to PCR in comparison with the boiling method.

**Conclusions:**

PCR method is a valuable, cost-effective and alternative tool for quick diagnosis of active tuberculosis in different clinical specimens.

## 1. Background

Tuberculosis (TB) represents a global burden and causes significant mortality, mostly in developing countries. It has been suggested that TB was the cause of death in 1.5 million people and infected almost 8.8 million new cases in 2010 (1). Therefore, an early diagnosis of TB is important for prevention of its spread. The gold standard test for diagnosis of TB is through the culture method (2). Culture method is not straightforward because isolation, identification (based on biochemical tests and phenotypic results) and drug susceptibility testing for this bacterium and other Mycobacterial isolates on solid media can take at least four to eight weeks or even longer. Furthermore, the turnaround time of *Mycobacterium tuberculosis* is high and the method is not always accessible (3). As a result, rapid and easy to perform methods are required to detect and differentiate the *Mycobacterium* (MYC) genus. 

The MYC includes members of *M. tuberculosis* complex (MTC) and species of non-tuberculosis mycobacteria (NTM). MTC strains are frequently associated with tuberculosis in developing countries while NTM infections are predominantly found in developed countries. Since many members of NTM are resistant to antibiotics used for tuberculosis treatment, rapid and more accurate differential diagnosis of mycobacterial infections is quite important (3, 4).

Molecular methods have the potential to detect both *M. tuberculosis* and non-tuberculous Mycobacteria directly from clinical samples (3). Recently, several PCR-based methods and DNA extraction protocols have been widely used for TB diagnosis in clinical laboratories (5-10). Although different DNA isolation methods have been developed, including enzymatic lysis using detergents, mechanical disruption, and heat lysis–based methods, few studies have assessed direct treating of crude clinical specimens in order to obtain Mycobacterial DNA without decontamination and/or more purification procedures (11, 12). To limit the steps, costs, amount of needed samples, risk of missing Mycobacterial DNA (through procedures prior to DNA isolation) and lower the possibility of cross contamination, we developed a new DNA isolation protocol. In addition, we assessed three different PCR methods using pan-Mycobacterial primers, IS6110 PCR assay and MTC/NTM multiplex PCR primers. 

A total of thirty different specimens of TB, from suspected patients referred to Tuberculosis Research Lab at Ghaem University Hospital, were used for this study. The samples underwent Ziehl-Nielsen (ZN) staining, culturing on LJ medium (gold standard) and PCR. DNA was extracted by boiling and buffer methods without more purification steps to save time, amount of samples and the risk of cross contamination.

## 2. Objectives

This study was carried out to compare culture and PCR methods for the diagnosis of *M. tuberculosis* in different clinical specimens using two different DNA extraction protocols.

## 3. Materials and Methods

### 3.1. Bacterial Isolates

Eight Mycobacterial isolates were collected and grown on Lewenstein Jensen (Merck, Germany) slant medium, which then formed clearly visible colonies. Six colonies belonged to *M. tuberculosis* complex and two were non-tuberculous Mycobacteria strains, which were further confirmed by phenotypic results and the PCR method.

### 3.2. Specimens

Thirty different clinical samples including urine (n = 4), gastric washout (n = 1), bronchoalveolar lavage (BAL) (n = 18), pleural fluid (n = 5), ascites tap (n = 1) and lung washout (n = 1) from tuberculosis suspected patients were collected from Ghaem University Teaching Hospital, Mashhad, Iran. 

### 3.3. Specimen Preparation

Each sample was used for three procedures, one for decontamination processing and two (1 mL each) for DNA extraction and PCR. Samples were decontaminated, homogenized and cultured on LJ medium by the Petroff technique (13). Two drops from concentrated and homogenized samples were used for indirect smear preparation. Smear preparation, ZN staining and slide reading were carried out according to the recommendations outlined in the Manual of Tuberculosis Bacteriology (14). Samples containing 1 mL body fluid were centrifuged at 1800g for 15 minutes; supernatants were discarded, and pellets were used for DNA isolation. 

### 3.4. Isolation of Mycobacterial DNA From Live Mycobacteria and Body Fluids

Two methods for DNA extraction were used. First *M. tuberculosis *DNA was extracted directly by the tissue digestion protocol. Four hundred microliters of the tissue digestion buffer (Tris-Cl, 100 mM, pH = 7.5; Tween-20, 0.05%) was added to each tube containing either pellets of clinical isolates or colonies. Then, 20 μL of 18.5 mg/mL solution of proteinase K (Fermentas, Germany) was added, agitated, and incubated at 55^o^C for 3 hours followed by 10 minutes of heating in boiling water in order to deactivate the action of proteinase K (15). The other protocol for DNA preparation was the simple boiling method. Colonies and pellets from clinical isolates were suspended in 400 µL of distilled water and heated for 10 minutes in a boiling water bath.

### 3.5. DNA Amplification

PCR was performed using four sets of specific primers to detect DNA of the members of *Mycobacterium* genus, *M. tuberculosis* complex and also non-tuberculosis Mycobacteria. A PCR protocol was performed using pan-Mycobacterial primers MYITSF (5´-GATTGGGACGAAGTCGTAACAAG-3´) and MYITSR (5´-AGCCTCCCACGTCCTTCATCGGCT-3´) (4) in a final 20 µL reaction volume comprised of 2 µL PCR 10X buffer (Genetbio, South Korea); 1.2 µL of 25 mM MgCl_2 _(Genetbio, South Korea); 0.6 µl of each of 10 pM oligonucleotide primers (Metabion International AG, Germany); 0.4 µL of 10 mM dNTPs (Genetbio, South Korea); 0.3 µl TaqDNA polymerase (Genetbio, South Korea); 12.9 µl Nuclease free water and 2 µL extracted DNA. The reaction was subjected to a PCR protocol as follows: 10 minutes at 94°C, followed by 35 cycles (94°C for 30 seconds, 62°C for 45 seconds, and 72°C for 45 seconds); cycles were followed by a final extension of 72°C for 10 minutes. 

A variable rpoB gene region from MTC or NTM was amplified using two sets of specific primers, including MTCF (5´-TACGGTCGGCGAGCTGATCCAAA-3´) and MTCR (5´-ACAGTCGGCGCTTGTGGGTCAAC-3´) and NTMF (5´-GGAGCGGATGACCACCCAGGACGTC-3´) and NTMR (5´-CAGCGGGTTGTTCTGGTCCATGAAC-3´) (4). The multiplex-touchdown PCR was optimized for efficient amplification of Mycobacterial DNA. The reaction mixtures in a final volume of 20 µL contained 2 µL PCR 10X buffer (Genetbio, South Korea); 1.2 µL MgCl_2 _(25 mM Genetbio, South Korea); 0.6 µL of each of 10 pM oligonucleotide primers of MTCF, MTCR, NTMF and NTMR (Metabion International AG, Germany); 0.4 µL of 10 mM dNTPs (Genetbio, South Korea); 0.2 µL of TaqDNA polymerase (Genetbio, South Korea); 11.8 µL of Nuclease free water and 2 µl of extracted DNA. The cycling parameters included an initial denaturation at 95°C for 5 mins; 1 cycle of 1 min at 95°C, 30 seconds at 69°C, and 1 min at 72°C; in subsequent 12 cycles the annealing temperatures were decreased by 1°C every cycle until the temperature reached 57°C; 22 cycles of 1 cycle of 1 min at 95°C, 30 seconds at 56°C, and 1 min at 72°C; followed by an additional cycle of 10 mins at 72°C. The IS6110-PCR assay was derived from the study of Espasa et al (12).

### 3.6. Detection of Amplified DNA

The amplified DNA products were visualized by UV illumination after agarose gel electrophoresis and green viewer staining.

### 3.7. Statistical Analysis

Laboratory data were analyzed by the SPSS v.20 software (SPSS Inc., Chicago, IL, USA). Sensitivity, specificity and accuracy of the PCR method were compared with that of the culture method.

## 4. Results

**Figure 1.**
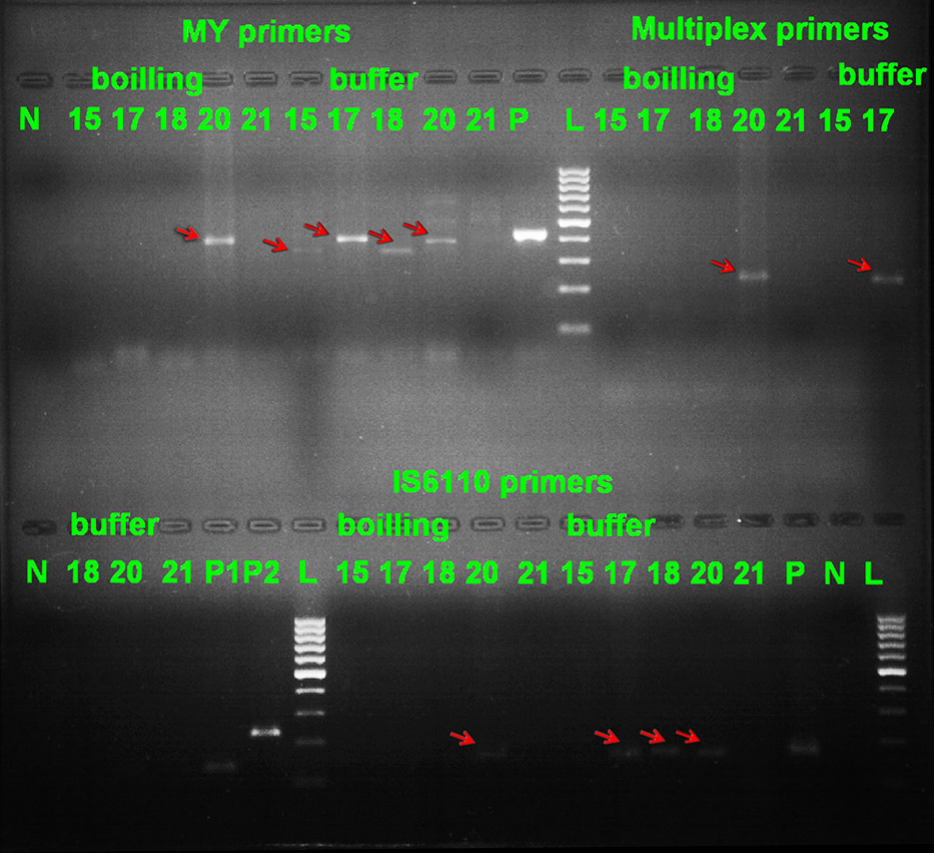
Representative Agarose Gel of PCR Products With All Four Sets of (MYITS, MTC, NTM and IS6110) Primers With Two DNA Extraction (Boiling and Buffer) Methods. Lane N: negative control; Lane 15, 17, 18, 21: negative samples with MYITS primers and boiling method; Lane 20 (around 400bp): positive sample with MYITS primers and boiling method; Lane 21: negative sample with MYITS primers and buffer method; Lane 15, 17, 18, 20 (around 350, 400, 350, 400 bp respectively): positive samples with MYITS primers and buffer method; Lane P: positive control for MYITS primers; Lane L: 100 bp DNA marker (Fermentas); Lane 15, 17, 18, 21: negative samples with MTC and NTM primers and boiling method; Lane 20 (around 235 bp): positive sample (MTC) with MTC and NTM primers and boiling method; Lane 15, 18, 20, 21: negative samples with MTC and NTM primers and buffer method; Lane 17 (around 235 bp): positive sample (MTC) with MTC and NTM primers and buffer method; Lane P1: positive control for *Mycobacterium tuberculosis* complex (MTC) strains; Lane P2: positive control for non-tuberculous mycobacteria (NTM) strains; Lane 15, 17, 18, 21: negative samples with IS6110 primers and boiling method; Lane 20 (around 150 bp): positive sample with IS6110 primers and boiling method; Lane 15, 21: negative samples with IS6110 primers and buffer method; Lane 17, 18, 20 (around 150 bp) positive samples with IS6110 primers and buffer method; Lane P: positive control for IS6110 primers; Lane N: negative control for IS6110 primers.

Out of thirty different clinical samples, 12 were positive by the culture method and 18 showed negative results. We used four sets of primers. A product of around 350-500 bp was obtained for the detection of MYC and products of around 235 bp and 136 bp were obtained from MTC and NTM strains, respectively. For detecting IS6110 insertion sequence, a product of around 150 bp was observed (Figure 1). 

Efficiency of bacilloscopy and PCR methods, in terms of sensitivity, specificity, positive predictive value (PPV), negative predictive value (NPV), likelihood ratio of a positive test result (LR+), likelihood ratio of a negative test result (LR-) and its accuracy in comparison to the culture method are shown in Table 1. In accordance to the binomial distribution, 95% confidence intervals (CIs) were estimated. Concordance between test results was assessed using the κ coefficients (Table 1). 

**Table 1.** Comparison of Results of Smear Microscopy and Different PCR-Methods Versus Culture on LJ Medium

	Culture (+) n=12	Culture (-) n=18	Sensitivity	Specificity	PPV^a^	NPV^a^	LR^a^ +	LR^a^ -	Accuracy	Kappa (P Value)
**Pan-myco/Boil (+/-) CI-95%**	5/7	3/15	41.67	83.33	62.5 (30.6-86.3)	68.18 (47.3-83.6)	2.5	0.7	66.67	0.265 (0.129)
**Pan-myco/Buffer (+/-) CI-95%**	8/4	4/14	66.67	77.78	66.67 (9.1-86.2)	77.78 (54.8-90.9)	3	0.43	73.33	0.444 (0.015)
**MTC/Boil (+/-) CI-95%**	1/11	0/18	8.33	100	100 (0.65- 100)	62.07 (44.0-77.3)	-	0.92	63.33	0.098 (0.213)
**MTC/Buffer (+/-) CI-95%**	5/7	0/18	41.67	100	100 (56.5-100)	72 (52.4-85.7)	-	0.58	76.67	0.462 (0.003)
**NTM/Boil (+/-) CI-95%**	1/11	1/17	8.33	94.44	50 (9.4- 90.5)	60.71 (42.4- 76.4)	1.5	0.97	60	0.032 (0.765)
**NTM/Buffer (+/-) CI-95%**	0/12	0/18	0	100	-	60 (42.3-75.4)	-	1	60	- (-)
**IS6110/Boil (+/-) CI-95%**	5/7	0/18	41.67	100	100 (56.5-100)	72 (52.4-85.7)	-	0.58	76.67	0.462 (0.003)
**IS6110/Buffer (+/-) CI-95%**	7/5	0/18	58.33	100	100 (64.6-100)	78.26 ) 58.1-90.3)	-	0.42	83.33	0.627 (0.0001)
**Smear (+/-) CI-95%**	9/3	3/15	75	83.33	75 (46.8-91.1)	83.33 (60.8-94.2)	4.5	0.3	80	0.583 (0.001)

^a^ Abbreviations: PPV, Positive predictive value; NPV, Negative predictive value; LR, Likelihood ratio of the 12 cases, which showed growth on LJ medium, 9 cases had positive results for smear microscopy.

The sensitivity of pan-Mycobacterial/buffer PCR (66.67%) was higher than other PCR methods such as NTM/buffer, NTM/boil, MTC\boil, MTC/buffer, IS6110\boil, pan-myco/boil and IS6110\buffer (0%, 8.33%, 8.33%, 8.33%, 41.67%, 41.67%, 41.67% and 58.33%, respectively) but the specificity was lower (77.78%) compared with other tests (the specificity of pan-myco/boil, NTM\boil, NTM/buffer, MTC\boil, MTC/buffer, IS6110\boil, and IS6110\buffer were 83.33%, 94.44%, 100%, 100%, 100%, 100% and 100%, respectively). The concordance rates between all tests were calculated and IS6110/buffer PCR method showed good agreement with the LJ culture method (κ = 0.627, P < 0.0001) and ZN staining smear microscopy (κ = 0.583, P < 0.001). The accuracy of IS6110/buffer PCR was higher (83.33%) than the other tests (Table 1). However, it was similar to smear microscopy (80%). IS6110 and MTC PCR using both buffer and boiling method showed 100% specificity and the NTM/buffer PCR method produced the same results. 

## 5. Discussion

Rapid identification of Mycobacterial infections is critical in clinical management of various diseases. It would determine the proper time for administration of antibiotics, the most suitable antibiotic, contact precautions and prophylaxis (4, 16, 17). Conventional diagnostic methods for detection of Mycobacterial infections are smear microscopy with ZN staining and culturing on LJ medium (4, 18). Despite the fact that microscopic smear examination is low-cost, rapid and easy to perform, it suffers from poor sensitivity and lack of distinctive specificity (19). Bacilloscopy normally requires more than one sample and does not differentiate the Mycobacterial genus. Culture is the gold standard test for diagnosis of TB. It has high specificity and its sensitivity is considered to be about 100 folds more than that of smear microscopy. The disadvantage of the culture method is its prolonged hands-on time and the need for gold standard laboratory infrastructure, which is limited to reference centers (18).

PCR-based assays have been used to detect Mycobacterial DNA with high sensitivity and specificity (3-9, 11, 12, 16, 19-21). In this study, we compared smear microscopy with three PCR-based methods using two different DNA extraction protocols. Culture method was regarded as the gold standard of TB diagnosis. 

Results showed that, bacilloscopy followed by homogenization and concentration by the Petroff technique showed 75% sensitivity and 83.33% specificity. Several studies showed similar results to our study (21-23). However, some studies reported a lower sensitivity of about 20% to 50% (20, 21, 24). In fact, the procedures used for bacilloscopy by such studies, were all rather indirect smear microscopy tests and the variety in their sensitivity may have been due to common laboratory errors. Lima et al. compared smear microscopy with the LJ culture method and found a kappa coefficient of 0.62, which was almost the same as that found by our study (0.583) (23).

We did not find NTM species among the specimens of our study. A reason for this observation could be the limited number of samples recruited in this study. Several studies have reported variable results regarding TB-PCR. Prakash et al. performed IS6110-PCR on purified DNA and reported sensitivity and specificity of 57% and 100%, respectively (25). In our study, the sensitivity of IS6110-buffer PCR on crude DNA was slightly higher (58.33%), however, the specificity was similar (100%). It may suggest that direct isolation of DNA by digestion buffer could be a valuable method to prevent the loss of bacterial DNA as opposed to the chloroform-phenol-isoamyl alcohol extraction method (25).

Use of commercial PCR kits on purified DNA before and after five days of brief-culture on LJ media resulted a sensitivity of 27.8% and 62.5%, respectively (26). It is expected that brief culturing, as part of the method proposed in this study, could increase the sensitivity of the test. Gupta et al. showed an overall sensitivity and specificity of 91.5% and 86%, respectively, for IS6110-PCR assay on purified DNA (27). In a study by Park et al. sensitivity and specificity of IS6110-Nested PCR on phenol-chloroform extracted DNA were reported to be 85% and 99%, respectively (28). An explanation for this observation could be the larger sample size (27) and also different PCR strategies (28).

Another report showed a PCR sensitivity of 85.7% and a specificity of 60% for diagnosis of *M. tuberculosis* in culture (22). Overall, our data showed that the buffer method is more efficient for direct DNA isolation compared with that of the boiling method. In the present study, pan-Mycobacterial/buffer PCR showed moderate to good sensitivity (66.67%) and specificity (77.78%). Kappa coefficient from comparison of PCR methods versus LJ culture showed a good agreement with IS6110/buffer PCR. It was also shown that IS6110/buffer PCR has the highest accuracy amongst PCR methods already discussed. Although PCR is a sensitive, specific, quick, straightforward and minimally invasive method for detection of *M. tuberculosis* in clinical samples, contamination of specimens with mycobacterial DNA from previous PCRs and/or contamination of samples during DNA isolation procedures may be the source of false positive results. 

This study compared conventional techniques and several PCR methods for diagnosis of tuberculosis. Since the sample size was too small, further studies are necessary to confirm, evaluate and improve the sensitivity, specificity and accuracy of the currently available tests. In conclusion, this study discussed a method, which is able to detect *M. tuberculosis* rapidly and directly in clinical samples. IS6110 PCR method might become a valuable, cost-effective and alternative tool for quick diagnosis of tuberculosis.
